# Oridonin Alleviates LPS-Induced Depression by Inhibiting NLRP3 Inflammasome *via* Activation of Autophagy

**DOI:** 10.3389/fmed.2021.813047

**Published:** 2022-01-12

**Authors:** Chunyan Li, Yuehua Zhu, Yuanyuan Wu, Meiyuan Fu, Yiling Wu, Yuehong Wu, Yinger Qiu, Hui Zhang, Mingxing Ding

**Affiliations:** ^1^Nursing Faculty, School of Medicine, Jinhua Polytechnic, Jinhua, China; ^2^Department of Psychiatry, Affiliated Jinhua Hospital, Zhejiang University School of Medicine, Jinhua, China; ^3^Medical Molecular Biology Laboratory, School of Medicine, Jinhua Polytechnic, Jinhua, China; ^4^Department of Psychiatry, The Second Hospital of Jinhua, Jinhua, China; ^5^Jinhua Center of Laboratory Animals, Jinhua Municipal Food and Drug Inspection Institute, Jinhua, China

**Keywords:** Oridonin, depression, autophagy, NLRP3 inflammasome, LPS

## Abstract

**Objective:** Oridonin (Ori) is a diterpene compound that has multiple biological properties. Here, our study was conducted to observe the therapeutic effect of Ori on depression as well as to uncover the mechanism.

**Methods:** Lipopolysaccharide (LPS)-induced depression models were established both in C57BL/6 mice and primary astrocytes, which were treated with Ori, autophagy agonist Rapamycin (Rap) and autophagy inhibitor 3-Methyladenine (3-MA). The depressive-like behaviors were assessed with behavioral tests. Autophagy was evaluated in the hippocampus and astrocytes by investigating autophagosomes under transmission electron microscope (TEM) and detecting LC3II/I, Beclin1 and P62 through western blotting. Astrocyte marker glial fibrillary acidic protein (GFAP) was investigated by immunofluorescence. NLRP3 inflammasome activation was evaluated by detecting IL-1β, NLRP3, ASC and Caspase-1 expression and reactive oxygen species (ROS) accumulation was quantified *via* DCFH-DA probe. Autolysosomes, autophagosomes and mitophagy were separately observed through mTag-Wasabi-LC3 plasmid, MitoTracker Deep Red staining, and TEM.

**Results:** Our results showed that Ori administration alleviated LPS-induced depressive-like behaviors and increased GFAP expression in the hippocampus. Furthermore, Ori treatment promoted autophagy activation and cell viability as well as weakened NLRP3 inflammasome activation and ROS accumulation both in LPS-induced mice and astrocytes. Ori promoted the autophagic flux unblocked through enhancing fusion of autophagosomes with lysosomes as well as enhanced mitophagy in LPS-treated astrocytes. The therapeutic effect of Ori was enhanced by Rap and weakened by 3-MA.

**Conclusion:** Collectively, our findings provided a promising antidepressant drug and uncovered that Ori alleviated LPS-induced depression by inhibiting NLRP3 inflammasome through activation of autophagy.

## Introduction

Depression represents a severe psychiatric disease, with a cluster of symptoms such as anhedonia, depressed mood, pessimism, and cognitive impairment (1). More than 350 million people suffer from depression worldwide (2). Depression, as one of the main mental diseases that cause mental and behavioral disorders, is an important factor leading to the global burden of disease (3). It is expected that depression will rise to the top factor resulting in the global burden of diseases by 2030 (4). Although the research on antidepressants, psychotherapy for depression and physical and mental treatment has made significant progress in recent years, there is still no completely satisfactory treatment strategy against depression (5).

It has been found that inflammatory response is implicated in the pathophysiology of depression (1). Increasing evidence suggests that various inflammatory factors are involved in the pathogenesis of depression (6). Among them, interleukin-1β (IL-1β) is closely related to abnormal mood and behaviors caused by stress (7). The cleavage and maturation of IL-1β precursor requires IL-1β converting enzyme, and IL-1β converting enzyme is an important component of the inflammasome (8). The inflammasome mainly modulates the function of the innate immune system as well as the mechanism of a series of inflammatory diseases (9). NLRP3 inflammasome is composed of NOD-like receptors, adaptor protein ASC and effector protein Caspase-1 (i.e., IL-1β converting enzyme), which can promote IL-1β, IL-18 and IL-33 shearing and maturation (10). NLRP3 inflammasome can be activated by a variety of exogenous or endogenous stress factors such as infection, reactive oxygen species (ROS), injury, and metabolites (11). NLRP3 inflammasome mediates the pathogenesis of depression *via* neuroinflammation (12), but it is not clear whether and how NLRP3 inflammasome is involved in the molecular mechanism of depression. Autophagy is an evolutionary conserved intracellular pathway, ensuring energy, organelle, and protein homeostasis by lysosomal degradation of damaged macromolecules or organelles (13). Emerging evidence suggests that autophagy is closely linked to stress-related diseases including depression (14). Studies have found that autophagy negatively modulates inflammasome activation; the occurrence of autophagy depends on specific inflammasome recognizers; inflammasomes are degraded through selective autophagy receptors; autophagy plays an important role in the secretion of the pro-inflammatory factor IL-1β (15–17). The basal level of autophagy regulates the activation of inflammasomes (18). If autophagy is inhibited, it leads to the accumulation of depolarized mitochondria, and then the leaked substances become endogenous inflammasome activators, such as mitochondrial DNA and ROS (19). Therefore, enhancing the level of autophagy can weaken inflammasome activation in depression.

Oridonin (Ori) is the main active ingredient of Rabdosia rubescens, which is widely used in Chinese medicine (20, 21). Ori has obvious anti-cancer activities such as inducing cell cycle arrest and apoptosis, inhibiting angiogenesis, etc. (22–24). But due to its relatively mild and imprecise mechanism of action, it greatly hinders the clinical application of Ori in cancer treatment. Ori can inhibit the activation of immune-related pathways, and inhibit the release of pro-inflammatory cytokines such as TNF-α and IL-6, which exhibits anti-inflammatory and protective effects in colitis, sepsis and neuroinflammation, etc. (25–27). Recent research has reported that Ori has the effect of suppressing depressive behaviors (28), but the detailed mechanisms are still unclear. Herein, we hypothesized that Ori may inhibit NLRP3 inflammasome activation by promoting autophagy, thereby having the function of alleviating depression.

## Materials and Methods

### Animals and Drug Administration

Healthy 8-week-old C57BL/6 mice with 18–22 g were provided from the Jinhua Center of Laboratory Animals (Zhejiang, China). All mice were raised at 18–22°C with 12 h light/12 h dark cycle, with free access to diet and water. All the experimental procedures were performed in line with the protocols approved by the Institutional Animal Care and Use Committee of School of Medicine, Jinhua Polytechnic (2019019). After 1 week of adaptive feeding, all mice were randomly separated into five groups (*n* = 6 each group): control group; lipopolysaccharide (LPS) group; LPS + Ori group; LPS + Ori + autophagy agonist Rapamycin (Rap) group; LPS + Ori + autophagy inhibitor 3-Methyladenine (3-MA) group. Ori (Sigma, USA) was dissolved in dimethyl sulfoxide (DMSO) and then diluted in 0.9% saline to a final concentration of 0.1% DMSO. Mice were given 20 mg/kg Ori by gavage (29) or were injected intraperitoneally by 3 mg/kg Rap or 10 mg/kg 3-MA daily for 7 consecutive days (30). Thirty min after the drug administration on the 7th day, mice were injected intraperitoneally with 1.2 mg/kg/day LPS or an equal amount of normal saline (31, 32). After 24 h of the LPS administration, all mice were subjected to behavioral tests before being perfused with glutaraldehyde and PFA, and were anesthetized by inhaling 4% halothane and subsequently sacrificed by cervical dislocation until the heartbeat and breathing stopped completely and the reflex disappeared. At last, brain tissues were collected and were stored at −80°C until before use.

### Sucrose Preference Test

As previously described (33), after 12 h of water deprivation, the mice were given pre-quantified 1% sucrose water and tap water at the same time. The position of the two tubes was changed after 6 h to prevent mice from having a position preference. After the mice drank freely for 12 h, the sucrose water pipe and the tap water pipe were collected and weighed. The sucrose preference rate (%) was determined according to the formula: sucrose preference rate (%) = sucrose water drinking amount (g)/(sucrose water drinking amount (g) + tap water drinking amount (g)) × 100%.

### Forced Swimming Test (FST)

As previously described (34), the mice were placed in glass beakers (height 24 cm, and diameter 13 cm) with a water depth of 14 cm and the water temperature was 22 ± 1°C. A video camera was used to record the behavior of the mice within 6 min. ForcedSwimScan^TM^ software (Clever Sys Inc., VA, USA) was used for analysis, and the immobility time of mice in the last 4 min was counted.

### Tail Suspension Test (TST)

As previously described (35), medical tape was utilized to fix the tail of mice on the top of the experiment box. A video camera was used to record the behavior of mice within 6 min. Tail Suspension Scan^TM^ software (Clever Sys Inc., VA, USA) was used for analysis. The time that mice's limbs and trunk were completely immobile in the last 4 min was counted.

### Transmission Electron Microscope (TEM)

After anesthesia with intravenous injection of pentobarbital sodium (5 mg/ 100 g), the mice were perfused by 2.5% glutaraldehyde as well as fixed *via* 4% paraformaldehyde. Then, secondary euthanasia was performed by cervical dislocation. Primary astrocytes were precipitated and fixed in 1% glutaraldehyde. About 1 mm^3^ hippocampus tissues or primary astrocytes were sectioned and incubated for 2 h at 4°C. After being postfixed by 1% osmium tetroxide, sections were treated with aqueous uranyl acetate. Afterwards, sections were dehydrated, embedded in epoxy resin as well as treated with lead citrate. Images were observed under a TEM (HT7700; Hitachi, Japan).

### Western Blotting

Brain tissues and primary astrocytes were lysed *via* RIPA on the ice lasting 30 min. The specimens were centrifuged at 12,000 g lasting 10 min at 4°C. The supernatant was gathered as well as protein concentrations were quantified utilizing BCA protein assay kits (P0009; Beyotime, Shanghai, China). Protein was separated *via* 12% SDS-PAGE, followed by being transferred onto a PVDF membrane. The membrane was sealed through 5% milk/TBST at room temperature for 1 h as well as incubated by primary antibody at 4°C overnight. Afterwards, the membrane was incubated by HRP-labeled goat anti-rabbit secondary antibodies (1/5000; SA00001-2; Proteintech, Wuhan, China) or HRP-labeled goat anti-mouse secondary antibodies (1/5000; SA00001-1; Proteintech, Wuhan, China) at room temperature lasting 1 h, and developed through ChemiDoc MP imaging system (Bio-Rad, USA). Primary antibodies included LC3 (1/2000; 14600-1-AP; Proteintech, Wuhan, China), Beclin1 (1/1000; 11306-1-AP; Proteintech, Wuhan, China), p62 (1/2000; 18420-1-AP; Proteintech, Wuhan, China), IL-1β (1/1000; 16806-1-AP; Proteintech, Wuhan, China), NLRP3 (1/1000; DF7438; Affinity, USA), ASC (1/2000; 10500-1-AP; Proteintech, Wuhan, China), Caspase-1 (1/1000; 22915-1-AP; Proteintech, Wuhan, China) and GAPDH (1/5000; ATPA00013Rb; AtaGenix, Wuhan, China). The results were quantified with ImageJ software (version 1.48; National Institutes of Health).

### Immunofluorescence (IF)

Hippocampus sections were permeated by 0.5%Triton X-100 at room temperature for 20 min and blocked by normal goat serum (C0265; Beyotime, Shanghai, China) at room temperature for 30 min. The sections were incubated by glial fibrillary acidic protein (GFAP) antibody (1/100; 16825-1-AP; Proteintech, Wuhan, China) at 4°C overnight and incubated by Alexa Fluor 488-conjugated AffiniPure Goat Anti-Rabbit IgG (H + L) (srbAF488-1; Proteintech, Wuhan, China) for 1 h. Images were captured under a BX53 fluorescence microscope (Olympus, Japan). The results were quantified with ImageJ software (version 1.48; National Institutes of Health).

### Real-Time Quantitative Polymerase-Chain Reaction (RT-qPCR)

RNA was extracted from brain tissues or primary astrocytes *via* Trizol (10606ES60; YEASEN, Shanghai, China) and reverse transcribed to cDNA in line with the following procedures: at 25°C lasting 5 min; at 42°C lasting 30 min and at 85°C lasting 5 min. Primer sequences included: GAPDH: 5′-TGTTTCCTCGTCCCGTAGA-3′ (forward) and 5′-GATGGCAACAATCTCCACTTTG-3′ (reverse), 116 bp; IL-1β: 5′-GTTCCCATTAGACAACTGC-3′ (forward) and 5′-GATTCTTTCCTTTGAGGC-3′ (reverse), 199 bp; NLRP3: 5′-CTCGCATTGGTTCTGAGCTC-3′ (forward) and 5′-AGTAAGGCCGGAATTCACCA-3′ (reverse), 153 bp; ASC: 5′-CAATGACTGTGCTTAGAGACATG-3′ (forward) and 5′-ACTTCTGTGACCCTGGCAATG-3′ (reverse), 175 bp; Caspase-1: 5′-CTGACTGGGACCCTCAAGTT-3′ (forward) and 5′-TCAACTTGAGCTCCAACCCT-3′ (reverse), 170 bp. RT-qPCR was presented through ABI 12K RT-qPCR instrument (ABI QuantStudio™ 12K Flex; ABI, USA). Relative mRNA expression was determined with 2^−ΔΔCt^ method.

### Immunohistochemistry (IHC)

Brain tissue specimen was fixed *via* 4% paraformaldehyde and embedded in paraffin. The section was cut into 4 μm. After xylene deparaffinization, hydration and antigen retrieval, the section was sealed through normal goat serum (C0265; Beyotime, Shanghai, China) lasting 20 min at room temperature. Then, the section was treated by primary antibodies against NLRP3 (1/100; NBP2-12446; Novus, USA) and IL-1β (1/50; Abs126104; absin, Beijing, China) at 37°C for 90 min and incubated by secondary antibody (1/100; SA00001-2/SA00001-1; Proteintech, Wuhan, China) lasting 30 min at room temperature. The color developing was presented *via* DAB reagent for 5 min. The nucleus was counterstained through hematoxylin (B600020; Proteintech, Wuhan, China) lasting 3 min as well as the section was washed by running water. After dehydration, the section was transparent and sealed with neutral gum. Images were acquired through an IX71 microscope (Olympus, Japan). The results were quantified with ImageJ software (version 1.48; National Institutes of Health).

### Primary Cell Culture and Treatment

Primary astrocytes were purchased from Procell company (CP-M157; Wuhan, China; https://www.procell.com.cn/), which were maintained in DMEM (SH30243.01B; Hyclone, USA) containing 10% fetal bovine serum (SH30084.03; Hyclone, USA), 100 U/mL penicillin, and 100 U/mL streptomycin in an environment of 37°C and 5% CO_2_. Astrocytes were treated with 0.1, 1.0 and 10 μM Ori. Astrocytes were induced by 2 μg/ml LPS to construct a depression *in vitro* model. Autophagy was activated by 25 ng/mL Rap and inhibited by 5 mM 3-MA.

### Cell Viability Assay

Counting kit-8 (CCK-8) detection kit (CK04; Dojindo, Shanghai, China) was utilized for detecting the cell viability. Primary astrocytes were seeded onto a 96-well plate (10^4^ cells/well). Each group was set to three repetitions. After incubation overnight, 20 μl of CCK-8 was added to each well and cells were incubated lasting 4 h. The absorbance values at 450 nm were examined with a microplate reader.

### Detection of ROS

ROS detection kit (CA1410; Solarbio, Beijing, China) was employed for examining ROS in primary astrocytes. DCFH-DA was diluted with serum-free culture medium according to 1:1000 to make the final concentration 10 μmol/L. After 48 h of drug treatment, astrocytes were incubated with 300 μl DCFH-DA at 37°C for 30 min. Then, astrocytes were washed with PBS to eliminate DCFH-DA which did not enter the astrocytes. In the culture plate, the slides with the climbed cells were immersed in PBS. Primary astrocytes were permeabilized through 0.5% Triton X-100 (T8787; Sigma, USA) at room temperature lasting 20 min as well as the slides were immersed in PBS. After absorbing the PBS with absorbent paper, normal goat serum (C0265; Beyotime, Shanghai, China) was added dropwise on the glass slide, and blocked for 30 min at room temperature. DAPI was added dropwise and incubated for 5 min in the dark to stain the nucleus, and the excess DAPI (D9542; Sigma, USA) was washed away with PBST. Following absorbing the liquid on the slide, the slides were mounted with a mounting solution containing anti-fluorescence quencher, and then observed and collected the images under a BX53 fluorescence microscope (Olympus, Japan).

### Transfection

Primary astrocytes were inoculated on the glass coverslip in a 24-well plate (1×10^6^ cells / well). 0.5 μg mTag-Wasabi-LC3 plasmids (800 ng/well) were transfected into astrocytes *via* Lipofectamine 2000 (Invitrogen, USA). The mTag-Wasabi-LC3 plasmid contained a red fluorescent protein mTagRFP, a green fluorescent protein mWasabi and an amino terminal of autophagy-labeled protein LC3 that was used for monitoring autophagic flux. In green/red merged images, yellow puncta indicated autophagosomes, and red puncta indicated autolysosomes. Following transfection for 6 h, LC3 fluorescent spot was acquired under a BX53 fluorescence microscope (Olympus, Japan). The vesicle of autolysosome was counted through Image-Pro Plus 6.0 software [National Institutes of Health (NIH)]. In total, 30 fields were randomly chosen for each sample.

### Measurement of Mitochondrial Activity

Primary astrocytes were seeded to poly-l-lysine-handled glass coverslips, followed by GFP-LC3 transfection. Mitochondria of astrocytes was stained by 100 nM MitoTracker Deep Red staining solution in the dark for 15 min. Images were captured by a BX53 fluorescence microscope (Olympus, Japan). Mitochondrial fluorescence intensity was quantified by ImageJ software (version 1.48; National Institutes of Health).

### Statistical Analysis

Statistical analysis was implemented with GraphPad Prism software (version 8.0.1). Each experiment was independently repeated three times. Data are displayed as mean ± standard deviation. Comparisons between two groups were analyzed with unpaired student's t test. Meanwhile comparisons between three or more groups were performed using one-way analysis of variance (ANOVA) followed by Turkey's *post-hoc* test. *P* < 0.05 indicated statistical significance.

## Results

### Ori Alleviates LPS-Induced Depressive-Like Behaviors

**Figure 1:**
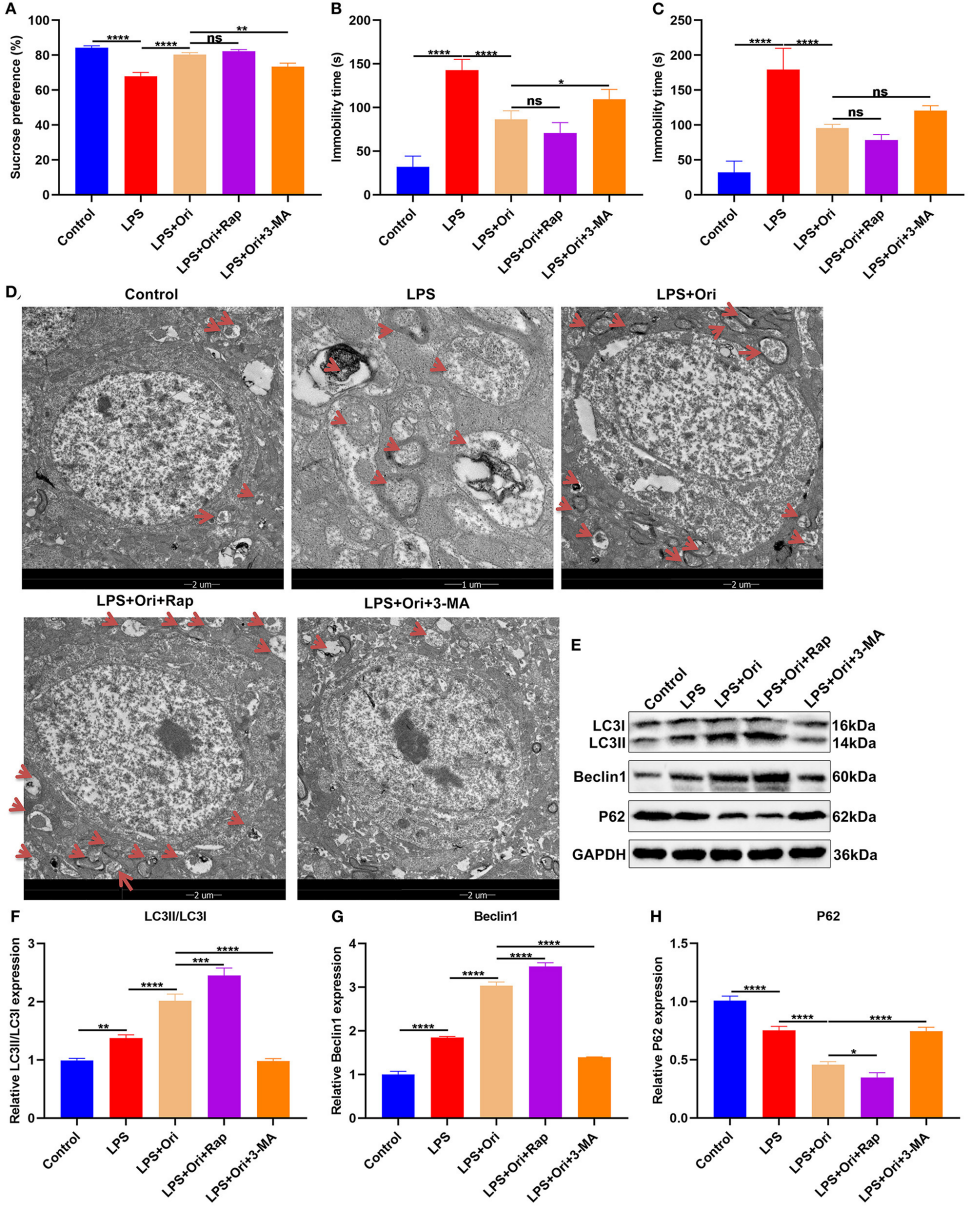
Effects of Ori, autophagy agonist Rap and inhibitor 3-MA on depressive-like behaviors in LPS-induced depression mouse models. **(A–C)** Sucrose preference, immobility time of FST and TST in each group. **(D)** TEM showing the size and number of autophagosomes in the hippocampus in each group. Red arrows, autophagosomes. Magnification, 5000× (1 μm) and 1700× (2 μm). **(E–H)** Western blotting for detecting LC3II/I, Beclin1 as well as P62 levels in the hippocampus of each group. *N* = 6 each group. *P* values were calculated with ANOVA with Turkey's *post-hoc* test. Ns, not significant; ^*^p < 0.05; ^**^p < 0.01; ^***^p < 0.001; ^****^p < 0.0001.

To determine whether Ori may prevent the development of LPS-induced depressive-like behaviors, this study preliminarily administrated mice with 20 mg/kg/day Ori and on the 7th day, 30 min after administration, the mice were injected intraperitoneally by 1.2 mg/kg LPS. Following 24 h of LPS injection, we evaluated depressive-like behaviors. The results showed that LPS significantly decreased sucrose preference (Figure 1A) as well as increased FST immobility (Figure 1B) and TST immobility (Figure 1C). On the contrary, Ori administration markedly elevated sucrose preference (Figure 1A) as well as reduced FST immobility (Figure 1B) and TST immobility (Figure 1C), indicative of the anti-depression roles of Ori. Under co-administration by autophagy inhibitor 3-MA, the therapeutic effects of Ori on sucrose preference and FST immobility were significantly weakened in LPS-induced mice. However, autophagy agonist Rap did not affect the roles of Ori on anti-depression.

### Ori Administration Enhances Autophagy Activation in the Hippocampus of LPS-Induced Depression Mouse Models

Autophagosomes with a vacuum-like bilayer structure enveloping the cell contents were investigated with TEM, which were pointed out by red arrows, as shown in Figure 1D. Compared with controls, the size and number of autophagosomes was increased in the hippocampus of LPS-induced depression mice. Both Ori and autophagy agonist Rap significantly enhanced the formation of autophagosomes and the converse results were found when treated by autophagy inhibitor 3-MA. Autophagy primarily involves three processes: formation of autophagosomes, transportation to the lysosomes as well as lysosomal degradation (36). We observed that LC3II/I and Beclin1 expression was significantly increased and P62 expression was markedly reduced in the hippocampus of LPS-induced depression mice (Figures 1E–H). Evidence suggests that enhancing the level of autophagy can treat LPS-induced depression (19). Herein, both Ori and autophagy agonist Rap distinctly enhanced LC3II/I and Beclin1 expression as well as significantly attenuated P62 expression, on the contrary when treated with autophagy inhibitor 3-MA. These data indicated that Ori administration enhanced autophagy activation in the hippocampus of LPS-induced depression mice.

### Ori Administration Protects Against LPS-Induced Astrocyte Damage *via* Autophagy Activation

**Figure 2:**
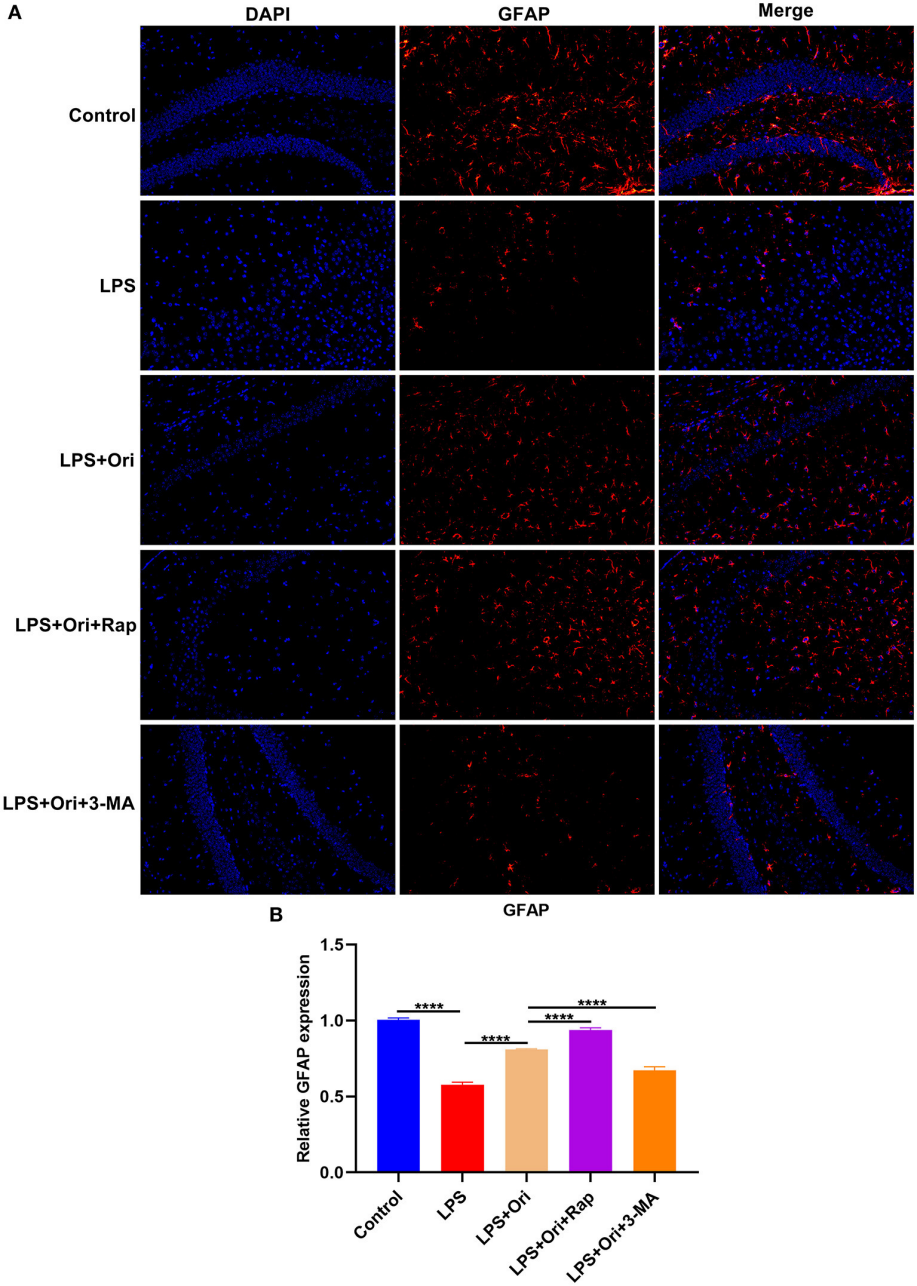
Effects of Ori, autophagy agonist Rap and inhibitor 3-MA on astrocyte activation in the hippocampus of LPS-induced depression mouse models. **(A)** Representative IF staining of GFAP in the hippocampus in each group. Magnification, 200× (50 μm). **(B)** Quantification of GFAP expression according to the IF staining results. *N* = 6 each group. *P* values were calculated with ANOVA followed by Turkey's *post-hoc* test. ^****^P < 0.0001.

To detect the involvement of astrocytes in LPS-mediated neuroinflammation, the expression of astrocyte activation marker GFAP was evaluated by IF staining. We observed that GFAP expression in the hippocampus was markedly reduced by LPS treatment, indicating that astrocytes were damaged (Figures 2A,B). Intriguingly, Ori administration prominently reduced the inhibitory effects of LPS on astrocytes. Hence, above findings supported that Ori administration protected against LPS-induced astrocyte damage. Furthermore, autophagy agonist Rap enhanced the protective effects of Ori on astrocytes and opposite results were observed when co-treatment with autophagy inhibitor 3-MA. This indicated that autophagy activation could be an indispensable factor for Ori-mediated astrocyte protection.

### Ori Administration Reduces NLRP3 Inflammasome Activation *via* Autophagy Activation in LPS-Induced Depression Mouse Models

**Figure 3:**
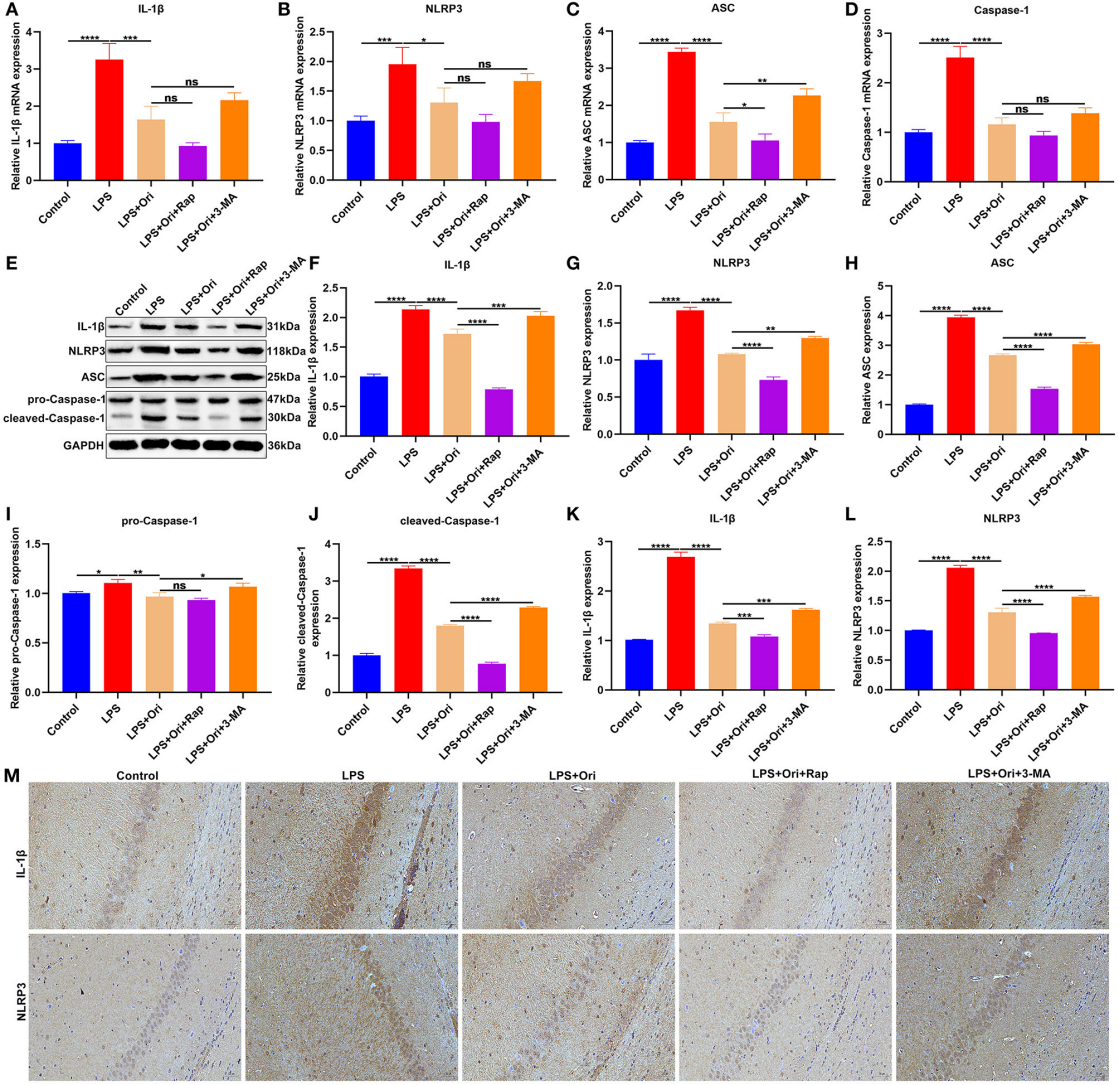
Effects of Ori, autophagy agonist Rap and autophagy inhibitor 3-MA on NLRP3 inflammasome in LPS-induced depression mouse models. **(A–D)** RT-qPCR for examining the relative mRNA levels of IL-1β, NLRP3, ASC as well as Caspase-1 in the hippocampus in each group. **(E)** Representative blots showing IL-1β, NLRP3, ASC, pro-Caspase-1, and cleaved-Caspase-1 in the hippocampus. **(F–J)** Quantitative analysis of IL-1β, NLRP3, ASC, pro-Caspase-1, and cleaved-Caspase-1 expression according to the western blotting results. **(K–M)** IHC showing the expression IL-1β and NLRP3 in the hippocampus. Magnification, 200× (50 μm). *N* = 6 each group. *P* values were calculated with ANOVA followed by Turkey's *post-hoc* test. Ns, not significant; ^*^p < 0.05; ^**^p < 0.01; ^***^p < 0.001; ^****^p < 0.0001.

We observed that the expression of NLRP3 inflammasome members IL-1β, NLRP3, ASC and Caspase-1 mRNAs was significantly up-regulated in the hippocampus of LPS-induced mice, indicative of the activation of NLRP3 inflammasome (Figures 3A–D). But Ori administration prominently reduced their mRNA expression in LPS-induced mice. Either autophagy agonist Rap or autophagy inhibitor 3-MA did not affect the inhibitory effects of Ori on the mRNA expression of IL-1β, NLRP3 and Caspase-1. Nevertheless, autophagy activation significantly enhanced the inhibitory effects of Ori on ASC expression and opposite results were investigated when autophagy was inhibited. As depicted in western blotting results, LPS treatment prominently induced the increase in IL-1β, NLRP3, ASC, pro-Caspase-1 and cleaved-Caspase-1 expression in the hippocampus (Figures 3E–J). Nevertheless, Ori administration markedly attenuated LPS-induced increase in IL-1β, NLRP3, ASC, pro-Caspase-1 and cleaved-Caspase-1 expression. Autophagy agonist Rap significantly strengthened the inhibitory effects of Ori administration on IL-1β, NLRP3, ASC and cleaved-Caspase-1 expression but did not affect pro-Caspase-1 expression in LPS-induced mice. Meanwhile, autophagy inhibitor 3-MA markedly attenuated the inhibitory effects of Ori on IL-1β, NLRP3, ASC, pro-Caspase-1 and cleaved-Caspase-1 expression. IHC staining was also presented for examining IL-1β and NLRP3 expression in the hippocampus. The results showed that LPS treatment markedly elevated IL-1β and NLRP3 expression in the hippocampus, which was prominently weakened by Ori administration (Figures 3K–M). The inhibitory effects of Ori on IL-1β and NLRP3 proteins were enhanced by autophagy agonist Rap, whereas were attenuated by autophagy inhibitor 3-MA. Hence, Ori administration significantly decreased NLRP3 inflammasome activation *via* autophagy activation in the hippocampus of LPS-induced depression mouse models.

### Ori Treatment Promotes Autophagy Activation of Astrocytes

**Figure 4:**
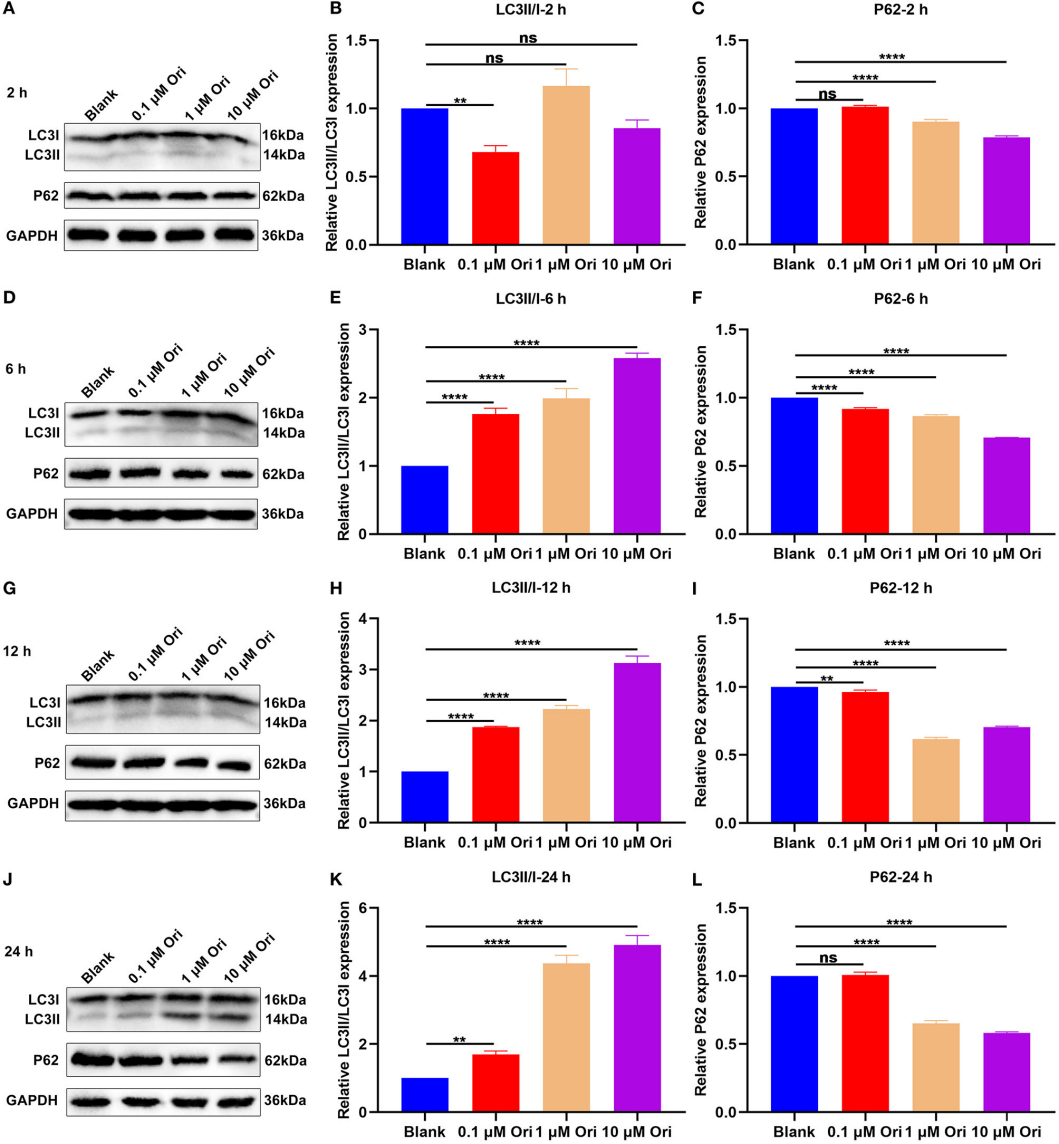
Effect of Ori treatment on autophagy activation in astrocytes. **(A–C)** Western blotting showing the expression of LC3II/I and P62 in astrocytes treated with 0, 0.1, 1 and 10 μM of Ori for 2 h. **(D–F)** Western blotting detecting the expression of LC3II/I and P62 in astrocytes treated with 0, 0.1, 1 and 10 μM of Ori for 6 h. **(G–I)** Western blotting quantifying the expression of LC3II/I and P62 in astrocytes treated with 0, 0.1, 1 and 10 μM of Ori for 12 h. **(J–L)** Western blotting quantifying the expression of LC3II/I and P62 in astrocytes treated with 0, 0.1, 1 and 10 μM of Ori for 24 h. *N* = 3 each group. *P* values were calculated with ANOVA followed by Turkey's *post-hoc* test. Ns: not significant; ^**^p < 0.01; ^****^p < 0.0001.

The effects of Ori treatment on autophagy were observed in primary astrocytes. Astrocytes were treated with 0, 0.1, 1 and 10 μM of Ori for 2, 6, 12 and 24 h. After treatment for 2 h, 0.1 μM Ori not 1 and 10 μM Ori significantly reduced the expression of LC3II/I in astrocytes, while 1 and 10 μM Ori not 0.1 μM Ori significantly decreased P62 expression (Figures 4A–C). Following treatment for 6 h, as the concentration of Ori increased, LC3II/I levels were gradually elevated and P62 levels were markedly reduced in astrocytes (Figures 4D–F). At 12 h of treatment, we found that LC3II/I expression exhibited significant increase gradually with an increase in concentration of Ori (Figures 4G,H). Meanwhile, Ori treatment significantly weakened the expression of P62, while P62 expression displayed the lowest expression under treatment with 1 μM Ori (Figures 4G,I). After treatment for 24 h, we observed that LC3II/I expression was gradually elevated with an increase in concentration of Ori (Figures 4J,K). Meanwhile, 1 and 10 μM Ori not 0.1 μM Ori markedly reduced P62 expression (Figures 4J,L). Taken together, our findings supported the stimulating effects of Ori treatment on autophagy activation in astrocytes. One μM Ori was selected as the optimal concentration and 6 h of treatment was selected as the optimal time.

### Ori Treatment Inhibits NLRP3 Inflammasome Activation and ROS Accumulation in Astrocytes

**Figure 5:**
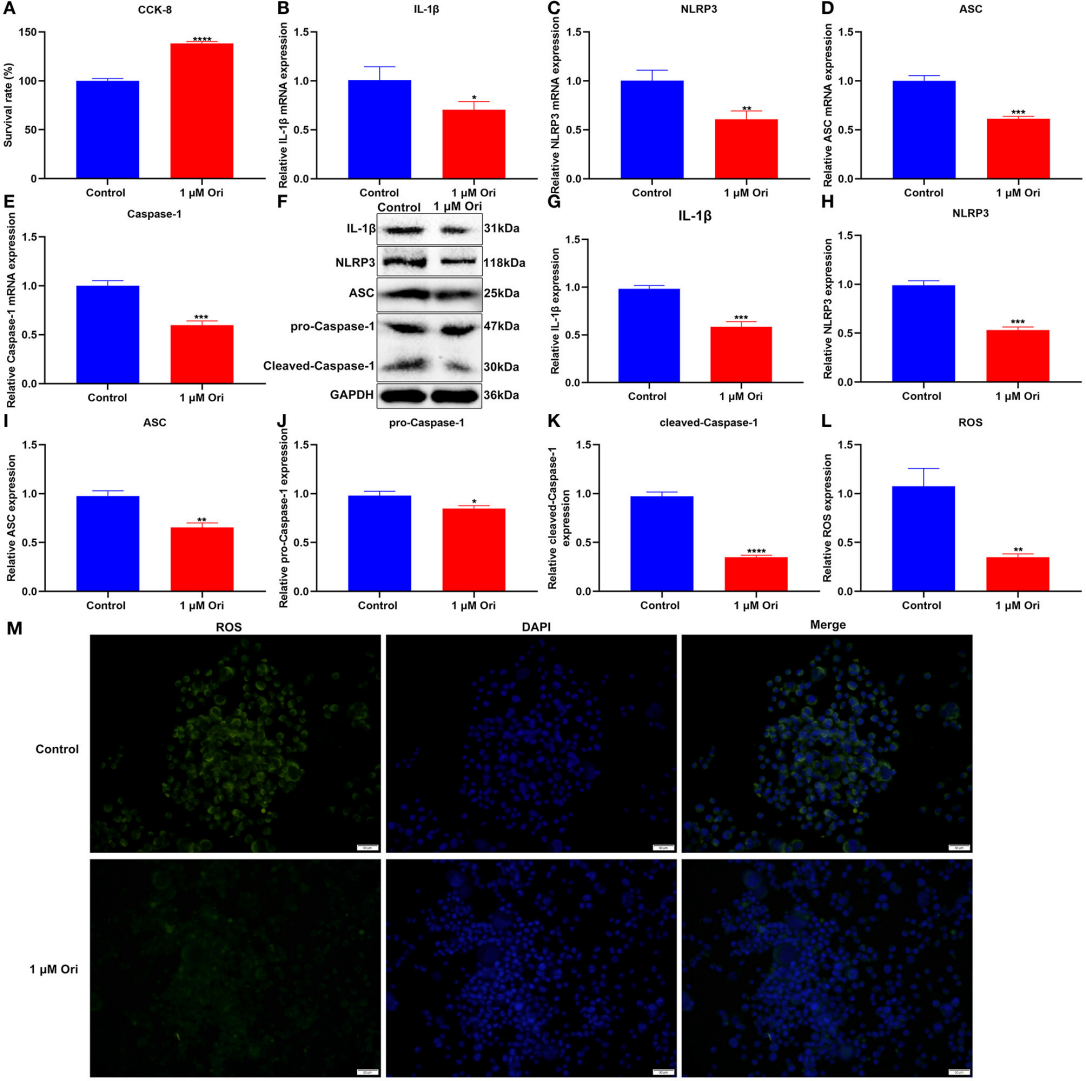
Effect of 1 μM Ori on NLRP3 inflammasome activation and ROS accumulation in astrocytes. **(A)** The cell viability of astrocytes treated with 1 μM Ori according to CCK-8 assay. **(B–E)** RT-qPCR of the relative mRNA expression of IL-1β, NLRP3, ASC and Caspase-1 in astrocytes treated with 1 μM Ori. **(F)** Representative blots showing IL-1β, NLRP3, ASC, pro-Caspase-1, and cleaved-Caspase-1 in astrocytes treated with 1 μM Ori. **(G–K)** Quantitative analysis of the expression of IL-1β, NLRP3, ASC, pro-Caspase-1, and cleaved-Caspase-1 according to the western blotting results. **(L)** Representative H2DCF-DA fluorescence staining of ROS in astrocytes treated with 1 μM Ori. **(M)** Quantitative analysis of the expression of ROS based on the H2DCF-DA fluorescence staining results. Magnification, 200× (50 μm). *N* = 3 each group. *P* values were calculated with student's *t* test. ^*^p < 0.05; ^**^p < 0.01; ^***^p < 0.001; ^****^p < 0.0001.

For confirming the protective roles of Ori on astrocytes, this study assessed cell viability through CCK-8 assay. We observed that 1 μM Ori distinctly promoted the viability of astrocytes (Figure 5A), indicative of the protective roles of Ori on astrocytes. The effects of Ori treatment on NLRP3 inflammasome activation were then investigated. The RT-qPCR results showed that 1 μM Ori markedly reduced the mRNA expression of IL-1β, NLRP3, ASC as well as Caspase-1 in astrocytes (Figures 5B–E). Meanwhile, we also investigated the decrease in IL-1β, NLRP3, ASC, pro-Caspase-1 as well as cleaved-Caspase-1 expression after treatment with 1 μM Ori (Figures 5F–K). These data were indicative of the inhibitory effects of 1 μM Ori on NLRP3 inflammasome activation in astrocytes. ROS may trigger NLRP3 inflammasome activation. Hence, we detected ROS accumulation by H2DCF-DA fluorescent probe in astrocytes. As expected, ROS expression was markedly weakened by 1 μM Ori treatment, suggesting that Ori could prevent ROS accumulation in astrocytes (Figures 5L,M).

### Ori Treatment Promotes Autophagy Activation in Astrocytes

**Figure 6:**
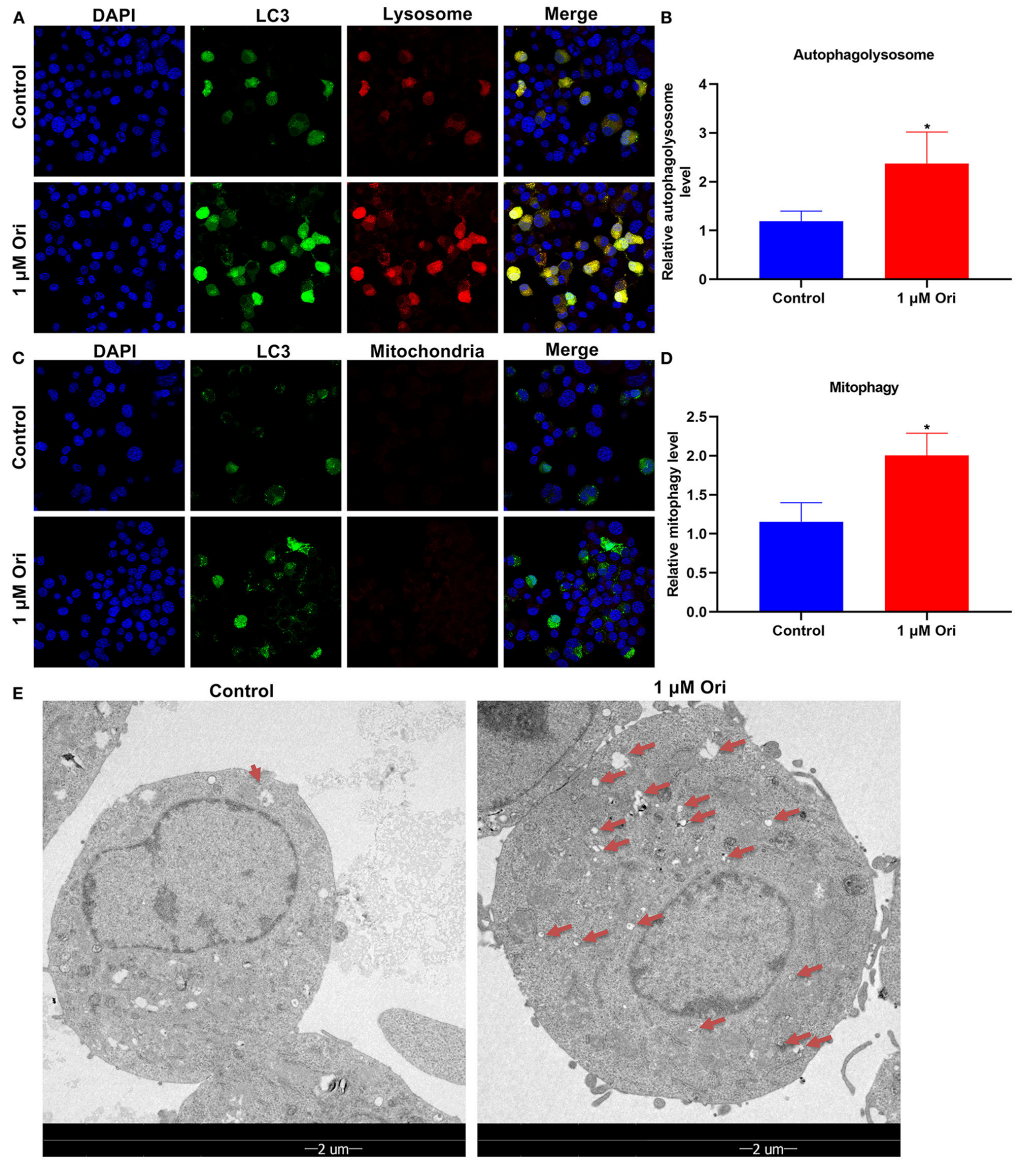
Effect of 1 μM Ori on autophagy activation in astrocytes. **(A)** IF staining for astrocytes transfected by mTag-Wasabi-LC3 plasmids. Magnification, 200× (50 μm). **(B)** Autolysosomes were counted in astrocytes treated with 1 μM Ori. **(C)** Astrocytes administrated by 1 μM Ori of which mitochondria were marked through MitoTracker Deep Red. Magnification, 200× (50 μm). **(D)** Mitophagy was quantified in astrocytes treated with 1 μM Ori. **(E)** TEM showing the morphology and number of autophagosomes in astrocytes treated with 1 μM Ori. Red arrows, autophagosomes. Magnification, 1700× (2 μm). *N* = 3 each group. *P* values were calculated with student's *t* test. ^*^P < 0.05.

Here, mTag-Wasabi-LC3 dual fluorescence staining was utilized for detection of the autolysosome and autophagosome accumulation. This study determined the effects of 1 μM Ori on autolysosomes and autophagosomes in astrocytes. In green/red merged images, yellow puncta indicated autophagosomes, while red puncta indicated autolysosomes. We found that 1 μM Ori distinctly induced autolysosome and autophagosome formation in astrocytes (Figures 6A,B). This showed that the acid-labile protein GFP-LC3 expressed by the fluorescent plasmid was degraded in the acidic environment inside the lysosome, indicating that the binding of autophagosomes and lysosomes was not hindered, and the autophagy flux remained unblocked after Ori was given. The effects of Ori on mitophagy were further observed. GFP-LC3 plasmid was transfected into astrocytes and MitoTracker Deep Red dye was used to label the mitochondria after 1 μM Ori treatment for 24 h. Each green fluorescent spot represented an autophagosome, and the red spot represented mitochondria in the cytoplasm. After 1 μM Ori treatment, we observed a significant increase in autophagosomes and its co-localization with mitochondria (Figures 6C,D), suggesting that Ori significantly promoted mitophagy in astrocytes. Autophagosomes were also monitored by TEM analysis. Our results showed that 1 μM Ori distinctly increased the number of autophagosomes in astrocytes (Figure 6E).

### Ori Treatment Protects Against NLRP3 Inflammasome Activation and ROS Accumulation *via* Autophagy Activation in LPS-Treated Astrocytes

**Figure 7:**
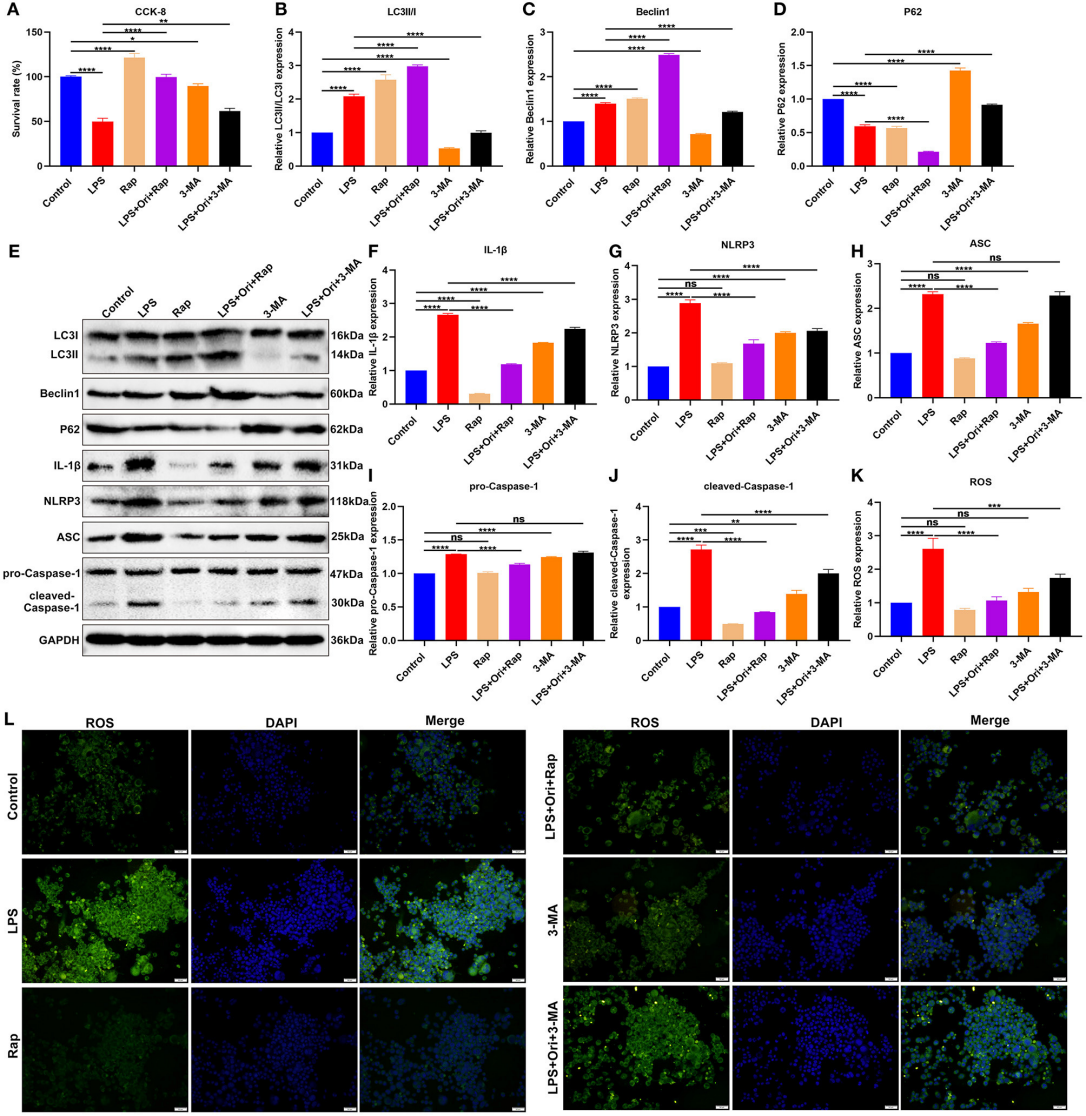
Effects of Ori, autophagy agonist Rap, and autophagy inhibitor 3-MA on NLRP3 inflammasome activation and ROS accumulation in LPS-treated astrocytes. **(A)** CCK-8 detecting the viability of astrocytes in each group. **(B–E)** Western blotting examining the expression autophagy markers LC3II/I, Beclin1 and P62 in astrocytes. **(F–J)** Western blotting testing the levels of NLRP3 inflammasome proteins IL-1β, NLRP3, ASC, pro-Caspase-1 as well as cleaved-Caspase-1 in astrocytes. **(K)** Quantitative analysis of the expression of ROS based on the H2DCF-DA fluorescence staining results. **(L)** Representative H2DCF-DA fluorescence staining of ROS in astrocytes. Magnification, 200× (50 μm). *N* = 3 each group. *P* values were calculated with ANOVA followed by Turkey's *post-hoc* test. Ns, not significant; ^*^p < 0.05; ^**^p < 0.01; ^***^p < 0.001; ^****^p < 0.0001.

Primary astrocytes were induced by LPS to construct depression *in vitro* models. Cell viability of primary astrocytes was markedly reduced by LPS treatment (Figure 7A). Compared with primary astrocytes, cell viability was significantly enhanced by autophagy agonist Rap and the opposite results were observed when treated with autophagy inhibitor 3-MA, indicating that autophagy activation may protect astrocytes against damage. One μM Ori and Rap co-treatment significantly ameliorated LPS-induced cell damage while 1 μM Ori administration markedly alleviated LPS-induced cell damage despite the existence of 3-MA. Thus, 1 μM Ori may protect against LPS-induced astrocyte damage *via* activating autophagy. Autophagy level was assessed through detecting the expression of LC3II/I, Beclin1 and P62. We observed that 1 μM Ori treatment markedly enhanced LC3II/I and Beclin1 expression and decreased P62 expression in LPS-treated astrocytes (Figures 7B–E). Meanwhile, 1 μM Ori treatment significantly ameliorated the inhibitory effects of 3-MA on autophagy in LPS-treated astrocytes. NLRP3 inflammasome activation was examined by detection of IL-1β, NLRP3, ASC, pro-Caspase-1 and cleaved-Caspase-1 in astrocytes (Figures 7F–J). Their expression was markedly increased by LPS and autophagy inhibitor 3-MA. Also, we observed that autophagy agonist Rap treatment significantly reduced IL-1β and cleaved-Caspase-1 levels in astrocytes. One μM Ori prominently reduced IL-1β, NLRP3, ASC, pro-Caspase-1 as well as cleaved-Caspase-1 expression in LPS-treated astrocytes under the existence of Rap or 3-MA. Above data suggested that 1 μM Ori weakened NLRP3 inflammasome activation through activating autophagy in LPS-treated astrocytes. ROS levels were increased in LPS-treated astrocytes, which was alleviated by 1 μM Ori treatment despite the existence of Rap or 3-MA (Figures 7K,L).

### Ori Treatment Enhances Autophagy in LPS-Treated Astrocytes

**Figure 8:**
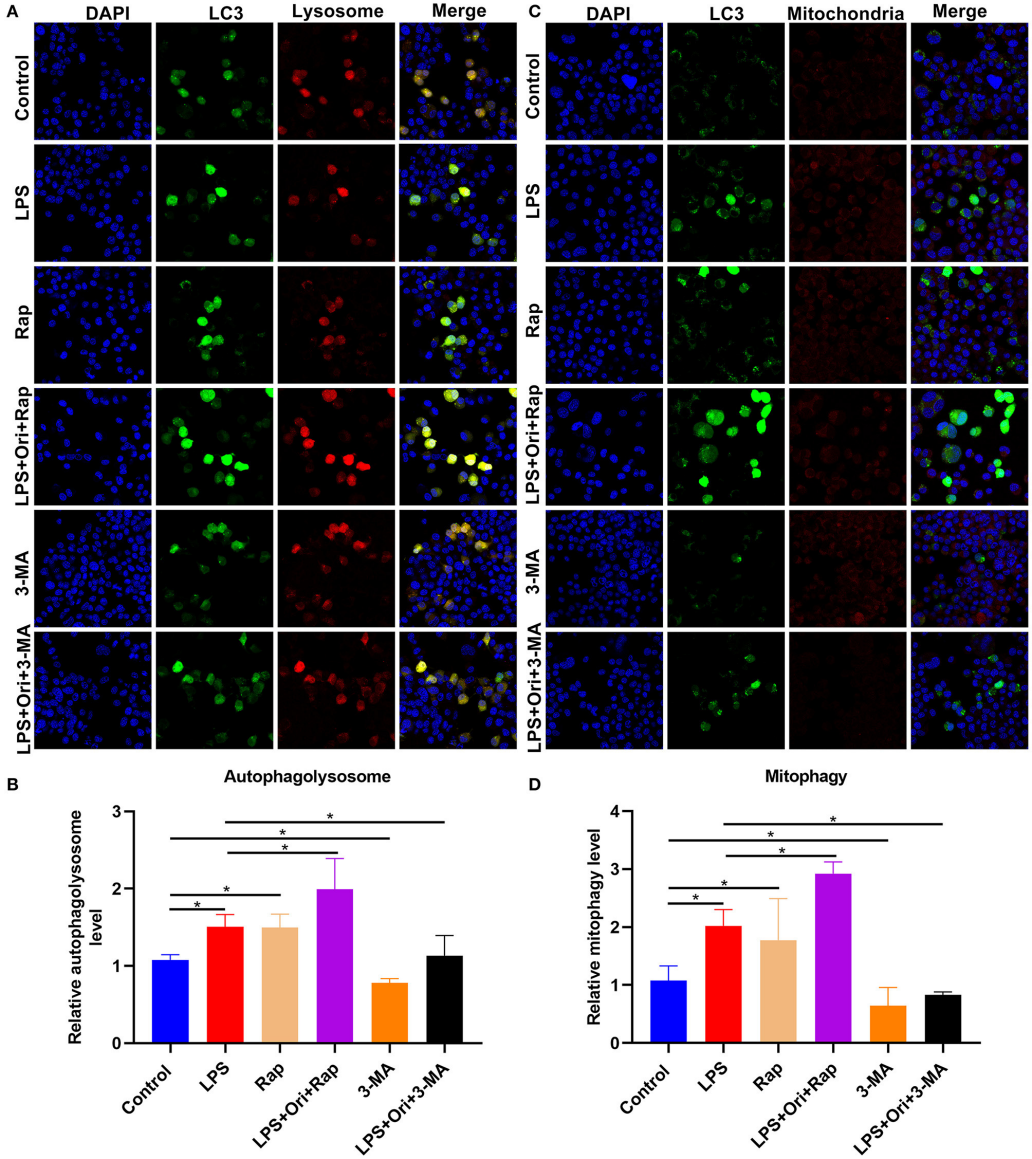
Effects of Ori, autophagy agonist Rap, and autophagy inhibitor 3-MA on autophagy activation in astrocytes. **(A)** IF staining showing astrocytes transfected by mTag-Wasabi-LC3 plasmids. Magnification, 200× (50 μm). **(B)** Quantification of autolysosomes in astrocytes. **(C)** Labeling mitochondria of astrocytes through MitoTracker Deep Red. Magnification, 200× (50 μm). **(D)** Quantification of mitophagy in astrocytes. *N* = 3 each group. *P* values were calculated with ANOVA followed by Turkey's *post-hoc* test. ^*^p < 0.05.

Through mTag-Wasabi-LC3 dual fluorescence staining, the autolysosome and autophagosome accumulation was detected in astrocytes. We observed that 1 μM Ori combined with Rap distinctly promoted autolysosome and autophagosome formation in LPS-treated astrocytes (Figures 8A,B). Furthermore, 1 μM Ori treatment combined with Rap significantly increased autophagosome formation and its co-localization with mitochondria (Figures 8C,D), suggesting that Ori significantly promoted mitophagy in LPS-treated astrocytes. However, 3-MA significantly weakened the stimulating effect of Ori on autophagy in LPS-treated astrocytes.

## Discussion

The cytokine theory, also known as the inflammatory response theory, has attracted more and more attention from researchers in the mechanisms of depression (37). The cytokine theory is based on the immunological changes in patients with depression. This theory believes that the inflammatory cytokines produced by the over-activated immune system play an important role in the pathogenesis of depression (38). At present, the cytokine theory has been confirmed by the research results of a series of clinical trials and animal experiments, so anti-inflammation therapy has increasingly become an emerging research direction in the prevention and treatment of depression (39). Here, we observed the therapeutic effects of Ori on depression by inhibiting NLRP3 inflammasome *via* activation of autophagy both in LPS-induced depression mice and LPS-treated astrocytes.

Our results showed that Ori treatment alleviated LPS-induced depressive-like behaviors in mice according to sucrose preference, FST and TST assays that have been widely applied as animal models for screening potential antidepressants. As previous evidence, autophagy inhibition induces depressive-like behaviors (40). Autophagy inhibitor 3-MA weakened the therapeutic effects of Ori on depressive-like behaviors. Furthermore, Ori treatment enhanced autophagy activation in the hippocampus of the LPS-induced depression model and LPS-treated astrocytes, indicating that Ori improved depressive-like behaviors through enhancing autophagy. Astrocytes are the richest glial cells in the mammalian brain, which act as a key regulator of cerebral development as well as survival and growth of normal neurons, formation of synapses, and action potential propagation (41). Astrocytes have special glial fibrils, which are composed of GFAP (42). Thus, GFAP can be used as a marker of astrocytes. Due to different dose, and different time line of administration of drugs compared with previous research (3), our results showed that GFAP expression was decreased in the hippocampus of mice after 1.2 mg/kg LPS administration, indicating that a single dose of LPS can damage astrocytes. We found that Ori improved the reduction of GFAP expression in the hippocampus of the LPS-induced depression model. Meanwhile, autophagy agonist Rap enhanced the promotion of Ori on GFAP expression, while autophagy inhibitor 3-MA abolished the protective role of Ori on astrocytes in the depression models, indicating that Ori can protect astrocytes by enhancing autophagy.

The inflammasome, a cytosolic multi-protein complex, includes NLRs, adaptor protein ASC and Caspase-1 (11). The NLRP3 inflammasome acts as a major pathway, which induces Caspase-1 activation as well as subsequent maturation of IL-1β (11). Our results for the first time showed that Ori treatment reduced the expression of NLRP3 inflammasome components including IL-1β, NLRP3, ASC and Caspase-1 both in the hippocampus of the LPS-induced depression models and LPS-treated astrocytes, which was enhanced by autophagy agonist Rap and weakened by autophagy inhibitor 3-MA. Previous evidence has shown that autophagy may suppress the NLRP3 inflammasome (16). ROS accumulation stimulates tissue inflammatory response and induces NLRP3 inflammasome activation (18). Our results demonstrated that Ori treatment weakened the production of ROS in LPS-treated astrocytes. Hence, Ori treatment could reduce LPS-induced NLRP3 inflammasome *via* enhancing autophagy. Under the TEM, we observed that Ori treatment significantly increased the formation of autophagosomes both in the hippocampus of the LPS-induced depression mice and LPS-treated astrocytes. To clarify what kind of autophagosome increased phenomenon belonged to, we used mTag-Wasabi-LC3 plasmid to transfect primary astrocytes. Our results showed that autophagosomes and autophagolysosomes were significantly increased in LPS-treated astrocytes after Ori treatment, indicating that autophagosome maturation was inhibited, that was, autophagosomes could not be combined with lysosomes made autophagosomes unable to degrade and accumulate. Mitophagy is a process of selectively removing damaged mitochondria through autophagy (43). Generally, the energy production of astrocytes is mainly based on mitochondrial oxidative metabolism in response to neuronal activity. Therefore, mitophagy as a selective autophagy plays a key role in determining the fate of astrocytes in depression. In this study, we found that Ori enhanced LPS-induced mitophagy in astrocytes, indicating that Ori promoted astrocyte autophagy flux and increased the clearance rate of damaged mitochondria in LPS-induced astrocytes. Damaged or non-functional mitochondria may release excessive ROS and cause cell damage (44). Our results showed that Ori treatment reduced the accumulation of ROS induced by LPS, indicating that Ori could eliminate excessive mitochondrial ROS accumulation, thereby reducing cell death caused by mitochondrial damage. Therefore, Ori promoted autophagy to eliminate damaged mitochondria and reduce cell death, ultimately improving the pathological changes of hippocampal astrocytes in depression.

Herein, LPS exposure was used to induce neuroinflammation and depressive-like behaviors both in mice and astrocytes. Previous research has showed that Ori can significantly ameliorate depressive-like behaviors of mice partly through regulating PPAR-γ/AMPA receptor signaling in the prefrontal cortex (28). Moreover, Ori ameliorates depressive-like behaviors *via* suppressing neuroinflammation and autophagy impairment in rat models with chronic unpredictable mild stress. However, the specific molecular mechanisms are lack of more experimental evidences. Herein, our study for the first time demonstrated that Ori treatment could reduce LPS-induced NLRP3 inflammasome *via* enhancing autophagy of astrocytes. However, there are a few limitations in our study. Firstly, we did not use a reference drug (antidepressant) like fluoxetine, though Ori possessed a remarkable anti-depressant property. Secondly, more experiments will be carried out for revealing the molecular mechanisms of Ori in treatment of depression. Thirdly, the therapeutic effect of Ori on depressive behaviors will be investigated in more studies.

## Conclusion

Taken together, our findings demonstrated that Ori could become a promising antidepressant drug. Ori may alleviate LPS-induced depression by inhibiting NLRP3 inflammasome through activation of autophagy both in the hippocampus of the LPS-induced depression model mice and LPS-treated astrocytes, which unveiled the new protective mechanism of Ori treatment in depression.

## Data Availability Statement

The original contributions presented in the study are included in the article/supplementary material, further inquiries can be directed to the corresponding author.

## Ethics Statement

The study was approved by the Ethics Committee of School of Medicine, Jinhua Polytechnic (2019019).

## Author Contributions

MD conceived and designed the study. CL, YZ, YuaW, and MF conducted most of the experiments and data analysis, and wrote the manuscript. YiW, YueW, YQ, and HZ conducted a small number of experiments and data analysis, and contributed to the writing of the manuscript. MD and CL confirm the authenticity of all the raw data. All authors reviewed and approved the manuscript.

## Funding

This work was funded by the Basic Public Welfare Research Program of Zhejiang Province (LGD20H090002) and the Science and Technology Project of Jinhua City in China (2019-4-070).

## Conflict of Interest

The authors declare that the research was conducted in the absence of any commercial or financial relationships that could be construed as a potential conflict of interest.

## Publisher's Note

All claims expressed in this article are solely those of the authors and do not necessarily represent those of their affiliated organizations, or those of the publisher, the editors and the reviewers. Any product that may be evaluated in this article, or claim that may be made by its manufacturer, is not guaranteed or endorsed by the publisher.

## Abbreviations

IL-1β: interleukin-1β
ROS: reactive oxygen species
Ori: Oridonin
LPS: lipopolysaccharide
Rap: Rapamycin
3-MA: 3-Methyladenine
FST: forced swimming test
TST: tail suspension test
TEM: transmission electron microscope
IF: immunofluorescence
GFAP: glial fibrillary acidic protein
RT-qPCR: real-time quantitative polymerase-chain reaction
IHC: immunohistochemistry
CCK-8: Counting kit-8.
